# Celastrol attenuates Th1- and Th2-driven skin inflammation in 2D and 3D *in vitro* models

**DOI:** 10.1038/s41598-026-47386-w

**Published:** 2026-04-02

**Authors:** Kirsten Reddersen, Bianka Morgner, Oliver Werz, Stefan Lorkowski, Dagmar Fischer, Jörg Tittelbach, Cornelia Wiegand

**Affiliations:** 1 Department of Dermatology, University Hospital Jena, Am Klinikum 1, 07747 Jena, Germany; 2 Department of Pharmaceutical/Medicinal Chemistry, Institute of Pharmacy, Friedrich Schiller University Jena, 07743 Jena, Germany; 3 Department of Biochemistry and Physiology of Nutrition, Institute of Nutritional Science, Friedrich Schiller University Jena, 07743 Jena, Germany; 4 Division of Pharmaceutical Technology and Biopharmacy, Friedrich-Alexander-Universität Erlangen-Nürnberg, 91058 Erlangen, Germany

**Keywords:** Celastrol, Inflammation, 2D inflammation model, HaCaT keratinocytes, Fibroblasts, 3D atopic dermatitis model, Diseases, Immunology

## Abstract

Celastrol, a bioactive compound from *Tripterygium wilfordii* Hook F, has been reported to exert potent anti-inflammatory effects through multiple signaling pathways. While its activity has been studied in various cell types and disease models, its effects in atopic dermatitis (AD) is limited to a few studies in mouse models and remains largely unexplored in *in vitro* cell models. In this study, anti-inflammatory effects of celastrol were assessed in Th1-driven 2D inflammation models of HaCaT keratinocytes and dermal fibroblasts (DF), and in a Th2-driven 3D AD skin model. Cytokine stimulation mimicked chronic inflammation. Celastrol was applied at sub-cytotoxic concentrations, and inflammatory markers were quantified on the mRNA (qPCR) and protein (ELISA) level. In 2D models, celastrol reduced interleukin (IL)-8 and IL-6 secretion in a concentration-dependent manner, with fibroblasts producing higher cytokine levels than keratinocytes. In 3D AD models, topical celastrol (10 µM) was well tolerated and markedly reduced secretion of IL-8, IL-6, IL-1α, and mRNA expression of *CXCL8*, *IL6*, *IL1B*, and *IL23A*, even under continuous Th2 stimulation. AD biomarker genes *CCL26*, *CA2*, and *NELL2* were unaffected, likely due to persistent cytokine exposure. Celastrol displayed strong anti-inflammatory activity in both Th1- and Th2-driven *in vitro* skin inflammation models, including a physiologically relevant 3D AD model. Its multitarget action support its potential as a topical treatment candidate for chronic inflammatory skin diseases.

**Supplementary Information:**

The online version contains supplementary material available at 10.1038/s41598-026-47386-w.

## Introduction

Inflammation is one of the most important physiological phases of wound healing, involving a range of cell types, including keratinocytes, fibroblasts, and immune cells such as macrophages, neutrophils, and lymphocytes^1^. A complex interplay of mechanical and chemical signals emanating from these cells or pathogens controls coordinated cell behaviour during the inflammatory phase^2^. However, when this physiological process is disrupted, pathological inflammatory mechanisms arise^3^. This can lead to the development of various inflammatory skin disorders, like atopic dermatitis (AD), psoriasis, hidradenitis suppurativa, or chronic wounds^3^. These conditions have a high prevalence, with AD being one of the most common.

AD is considered one of the most common inflammatory skin diseases in childhood, with its prevalence having tripling over the last 30 years^4^. In industrialized countries, approximately 15–30% of children and 2–10% of adults are affected, with increasing rates also observed in developing countries^4^. Inflammatory skin diseases represent a growing problem for public and global health. Therefore, there is an urgent need for alternative anti-inflammatory treatment strategies that are more effective and better tolerated^3–5^. Understanding the molecular mechanisms underlying inflammatory pathways using preclinical models contributes to the improvement of therapeutic approaches^3^.

Bioactive ingredients from Traditional Chinese Medicine (TCM) have emerged as key compounds in current biomedical research. Celastrol, a highly bioactive pentacyclic triterpene, can be isolated from various plants used in TCM, including *Tripterygium wilfordii* Hook F, *Celastrus orbiculatus*, *Celastrus aculeatus*, *Celastrus reglii*, *Celastrus scandens*, and others belonging to the *Celastraceae* family^6,7^. In TCM, extracts from these plants are traditionally used to treat various autoimmune and inflammatory diseases such as rheumatoid arthritis, systemic lupus erythematosus, autoimmune hepatitis, and Crohn’s disease^6,8,9^. Numerous *in vitro* and *in vivo* studies have demonstrated the broad pharmacological efficacy of celastrol, including anti-inflammatory, anti-tumor, anti-obesity, immunosuppressive, and antioxidant effects^7–10^. Beyond its effects on rheumatoid arthritis, celastrol has also demonstrated anti-inflammatory activity in other diseases, including ulcerative colitis, diabetes, obesity, and psoriasis^6,8,11^. The models to investigate these anti-inflammatory effects of celastrol have primarily been animal models^7,12–14^ but also *in vitro* cell models like HUVECs^10^, macrophages^15,16^, keratinocytes^12,17^, and 3D psoriasis skin models^17,18^.

The aim of this study is to investigate the anti-inflammatory potential of celastrol using skin inflammation models of varying complexity that represent different inflammatory conditions. The models used include a Th1-driven 2D inflammation model based on HaCaT keratinocytes and dermal fibroblasts as well as a Th2- dominant 3D AD disease model.

## Results

### Cytotoxicity and anti-inflammatory effects of celastrol in 2D keratinocyte and fibroblast inflammation models

**Fig. 1:**
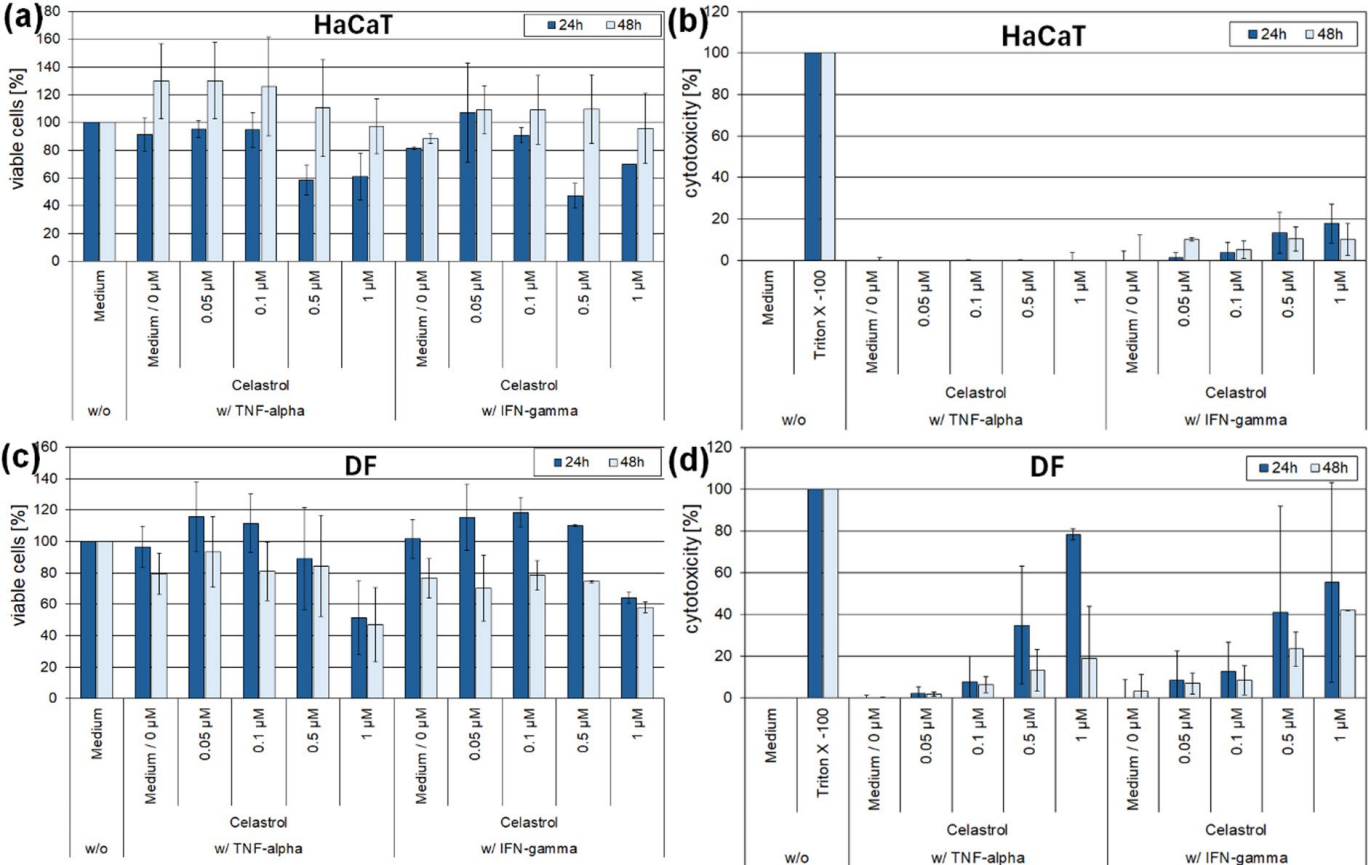
Determination of the cytotoxicity of celastrol in the 2D inflammation model of HaCaT keratinocytes (**a**,** b**) and primary human dermal fibroblasts (DF) (**c**,** d**) under stimulation with TNF-α or IFN-γ. Viability was measured using a luminometric ATP assay (**a**,** c**). Cytotoxicity was determined by measuring the activity of LDH in the cell supernatants (**b**,** d**). Medium without inflammatory stimulation served as negative control. Triton X-100 served as positive control in the LDH assay. Medium with 0.02% DMSO (0 µM celastrol) served as solvent control.

Incubation of tumor-necrosis-factor-α (TNF-α)- or interferon- γ (IFN-γ)-stimulated HaCaT keratinocytes with celastrol resulted in a reduction of cell viability after 24 h at concentrations of 0.5 and 1 µM using the ATP assay (Fig. 1a). After 48 h, the cytotoxic effect of celastrol at these concentrations was less pronounced. No release of the cytotoxicity marker LDH was detected in TNF-α-stimulated HaCaT keratinocytes, and only to a slight increase was observed in IFN-γ-stimulated HaCaT keratinocytes treated with 0.5 or 1 µM celastrol after 24 h (Fig. 1b). In dermal fibroblasts, incubation with 1 µM celastrol reduced cell viability after 24 and 48 h under TNF-α or IFN-γ stimulation (Fig. 1c). LDH release was detected at 0.5 µM celastrol after 24 h in TNF-α-stimulated fibroblasts, and at 0.5 and 1 µM celastrol after 24 and 48 h in IFN- γ-stimulated fibroblasts (Fig. 1d).

**Fig. 2:**
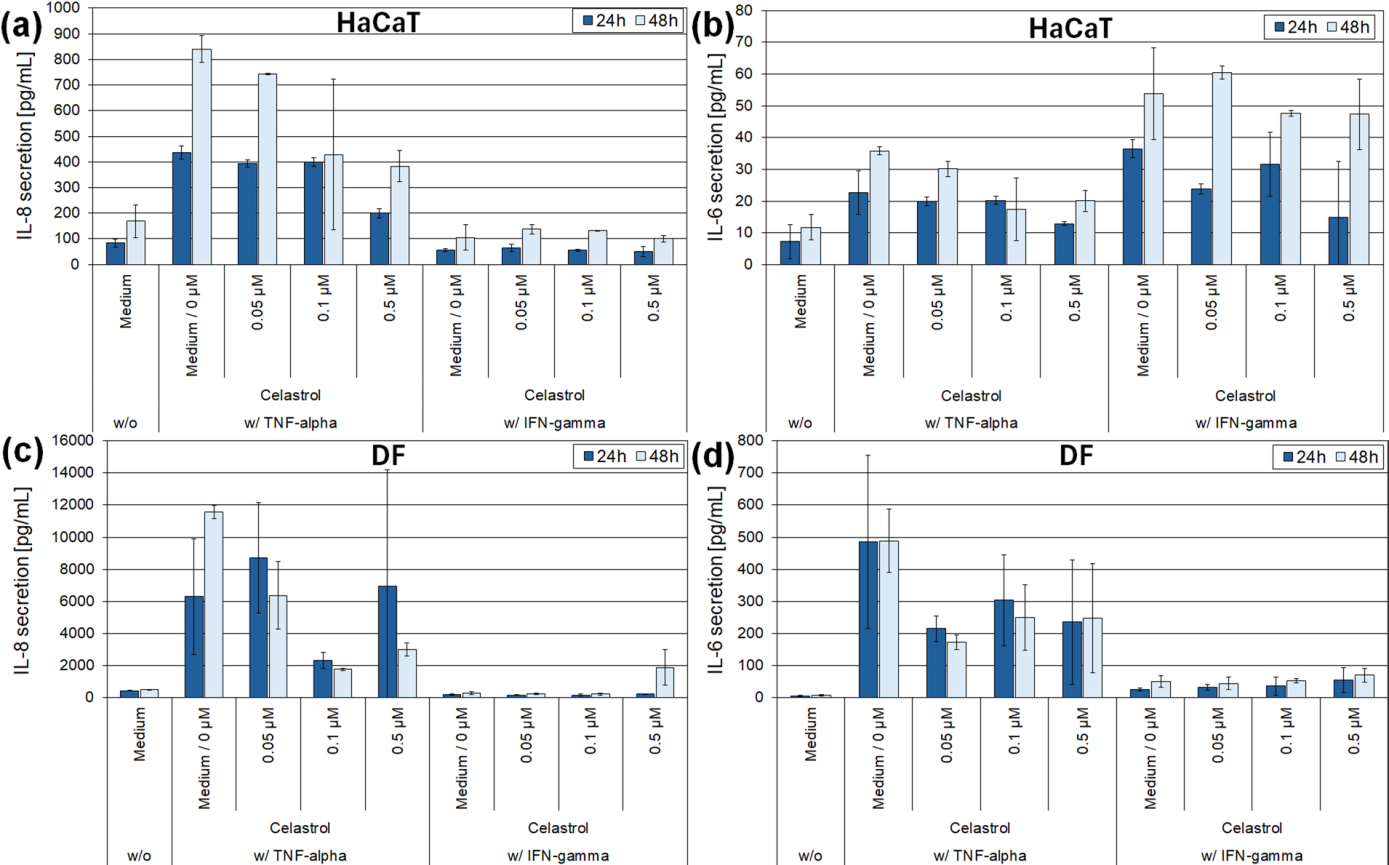
Determination of IL-6 and IL-8 secretion after celastrol treatment in the 2D inflammation model of HaCaT keratinocytes (**a**,** b**) and primary human dermal fibroblasts (DF) (**c**,** d**) under stimulation with TNF-α or IFN-γ. Cytokine levels were measured using ELISA-kits. Medium without inflammatory stimulation served as negative control. Medium with 0.02% DMSO (0 µM celastrol) served as solvent control.

Due to cytotoxic effects of celastrol in the 2D inflammation models, subsequent investigations of its anti-inflammatory effect were conducted without the highest cytotoxic concentration of 1 µM with celastrol concentrations up to 0.5 µM. Stimulation of HaCaT keratinocytes with TNF-α induced a strong secretion of the pro-inflammatory cytokines IL-8 and IL-6 (Fig. 2a, b). Simultaneous incubation with celastrol resulted in a concentration-dependent reduction of IL-8 and IL-6 after 24 and 48 h. Stimulation of HaCaT keratinocytes with IFN-γ did not affect IL-8 secretion (Fig. 2a) but led to a stronger IL-6 secretion compared to TNF-α stimulation (Fig. 2b). Incubation with increasing concentrations of celastrol reduced IL-6 secretion in a concentration-dependent manner, with the strongest downregulatory effect observed at 0.5 µM celastrol after 24 h (Fig. 2b).

Stimulation of dermal fibroblasts with TNF-α resulted in a strong secretion of the pro-inflammatory cytokines IL-8 and IL-6 compared to the unstimulated control (Fig. 2c, d). The levels of secreted cytokines were 10–20 times higher than those observed in HaCaT keratinocytes stimulated with the same concentration of TNF-α (Fig. 2). Simultaneous incubation of TNF-α-stimulated fibroblasts with celastrol reduced IL-8 and IL-6 secretion in a concentration-dependent manner (Fig. 2c, d). The most pronounced reduction in IL-8 secretion was observed at 0.1 µM celastrol, whereas the reduction of IL-6 secretion was consistent across all test concentrations. Stimulation of dermal fibroblasts with IFN-γ had no effect on IL-8 secretion and only slightly increased IL-6 secretion (Fig. 2c, d). Increasing concentrations of celastrol did not display a detectable anti-inflammatory effect in IFN-γ-stimulated dermal fibroblasts (Fig. 2c, d).

### Regeneration of the 3D AD model

**Fig. 3:**
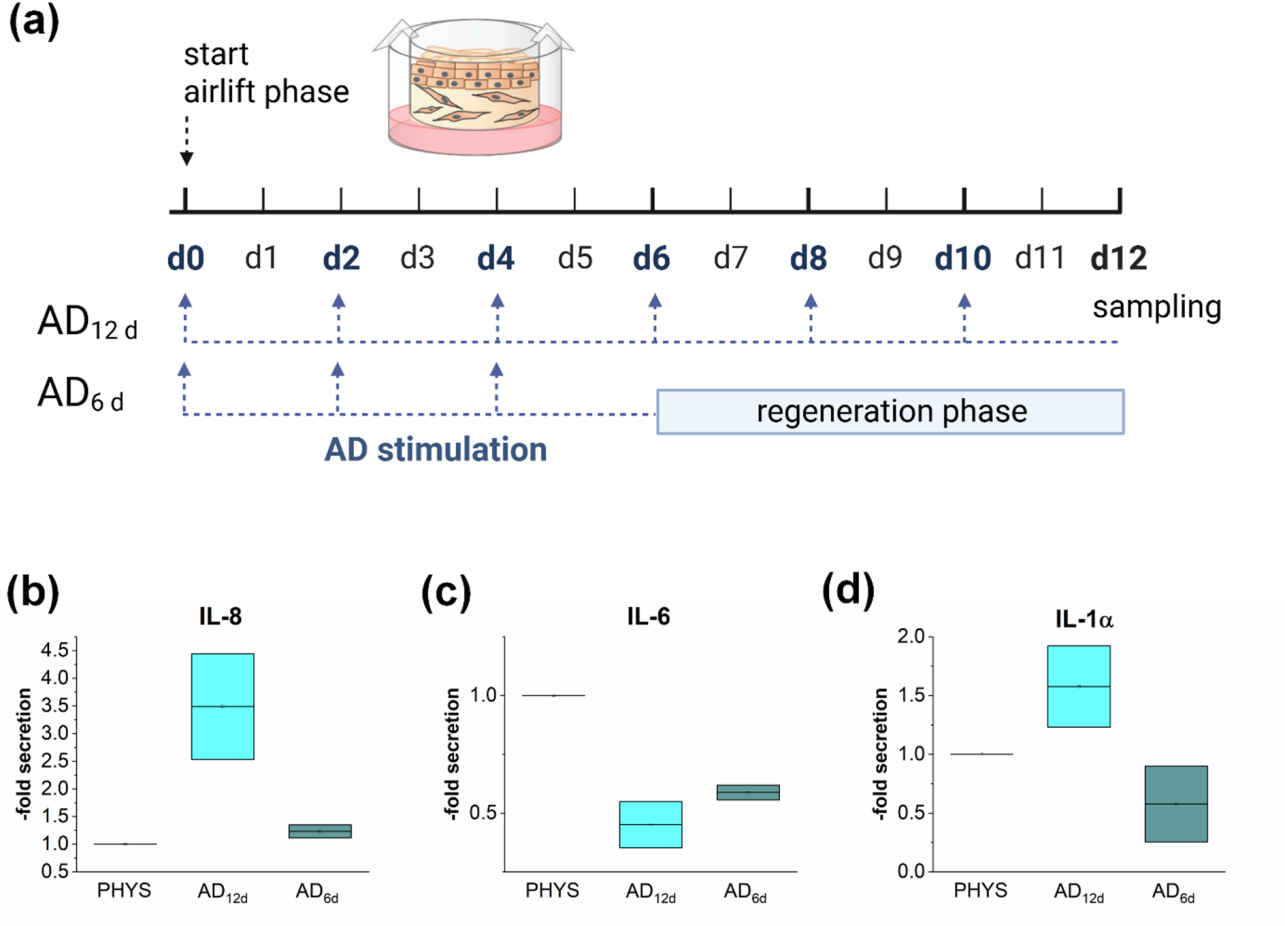
Regeneration potential of the 3D AD skin models. Samples were stimulated to induce characteristics of AD by adding a Th2 cytokine mix to the medium on days 0, 2, 4, 6, 8, and 10 during a 12 day airlift period (AD_12d_). To test the regeneration ability of the disease models, the Th2 cytokine mix was fed on days 0, 2, and 4 of the airlift period, on days 6, 8, and 10 medium without the Th2 cytokine mix was fed (AD_6d_) (**a**) Created in BioRender. Reddersen, K. (2025) https://BioRender.com/q43s554. Cytokine levels of IL-8 (**b**), IL-6 (**c**), and IL-1α (**d**) were measured using ELISA-kits. Skin models without the Th2 cytokine priming during the airlift period served as control (PHYS).

A prerequisite for using *in vitro* disease models as predictive tools for treatment studies is their ability to revert to a physiological phenotype. The AD phenotype in the 3D skin models was induced by Th2 cytokine priming during the airlift phase of 12 days. To assess the regenerative potential of the AD models, a 6-day stimulation phase with the Th2 cytokine mix was followed by 6 days without the pathological cytokine mix to allow regeneration (Fig. 3a). Fully stimulated AD_12d_ models secreted elevated levels of IL-8 and IL-1α and reduced levels of IL-6 compared to the physiological control (Fig. 3b, c, d). In the regenerated AD_6d_ models, the inflammatory reaction was reversed, with IL-8 and IL-1α cytokine release returning to levels comparable to the physiological control (Fig. 3b, d). IL-6 levels in the regenerated AD_6d_ models were increased compared to the fully stimulated AD_12d_ models, but had not yet fully returned to physiological levels (Fig. 3c).

**Fig. 4:**
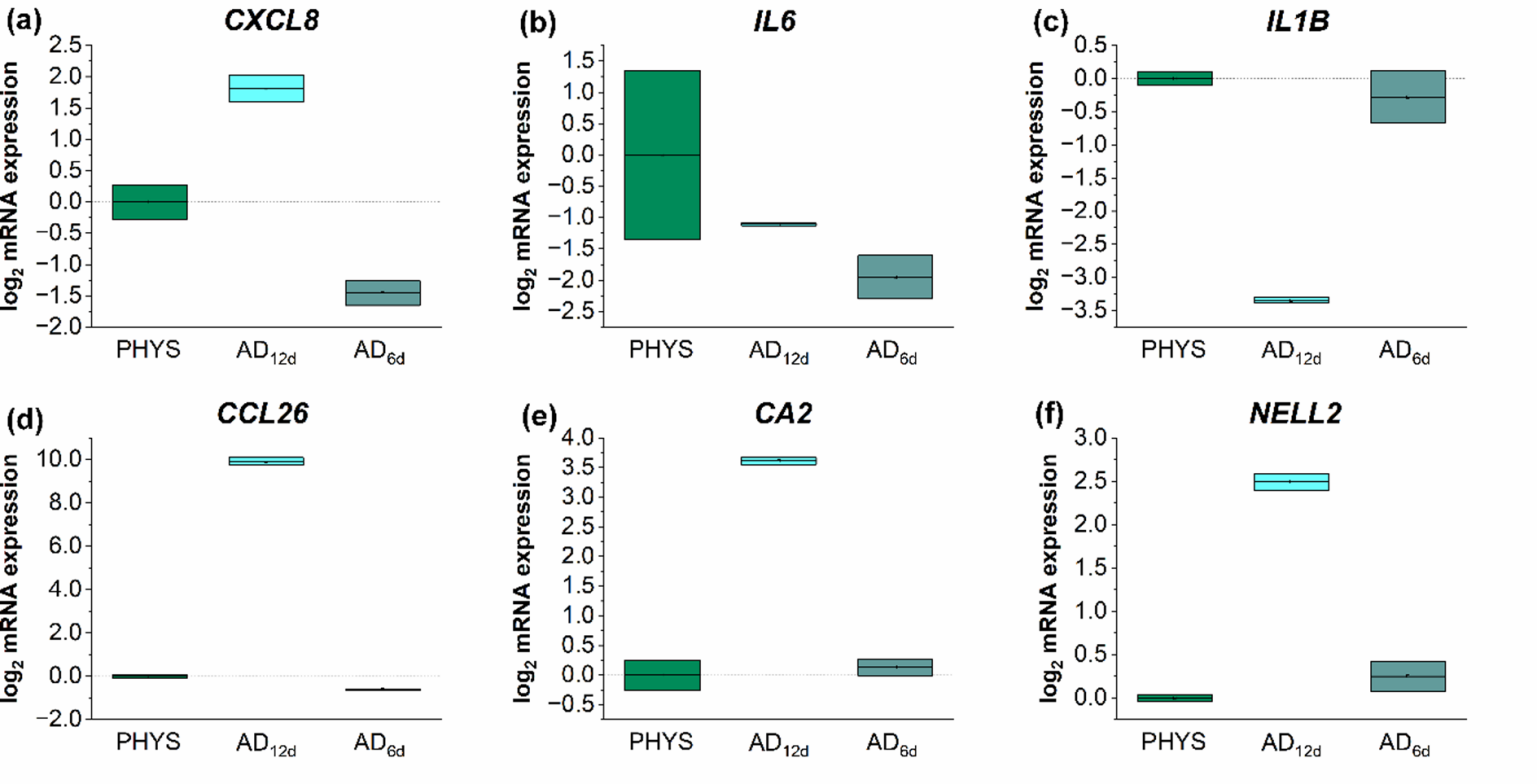
Determination of the relative mRNA expression of *CXCL8* (**a**), *IL6* (**b**), *IL1B* (**c**), *CCL26* (**d**), *CA2* (**e**), and *NELL2* (**f**) in the regeneration experiment of the 3D AD model compared to the physiological control. Samples were stimulated to induce characteristics of AD by adding a Th2 cytokine mix to the medium on days 0, 2, 4, 6, 8, and 10 during a 12 day airlift period (AD_12d_). To test the regeneration ability of the disease models, the Th2 cytokine mix was fed on days 0, 2, and 4 of the airlift period, on days 6, 8, and 10 medium without the Th2 cytokine mix was fed (AD_6d_). Skin models without the Th2 cytokine priming during the airlift period served as control (PHYS). Gene expression was measured using RT-qPCR. Values were plotted as log2 changes with log_2_ (2) = 1 presenting a doubled upregulation and log_2_ (0,5)=−1 presenting a downregulation by factor 2.

Additionally, the regenerative potential of the AD models was evaluated at the transcriptional level. Fully stimulated AD12d models displayed increased mRNA expression levels of the pro-inflammatory marker CXCL8 and reduced expression of IL6 and IL1B compared to the physiological control, (Fig. 4a, b, c). After a regeneration phase of 6 days, CXCL8 mRNA expression in AD6d models returned to levels comparable to those of the physiological control, (Fig. 4a). However, the mRNA expression of IL6 in AD6d models was lower than in AD12d models and remained below physiological levels, (Fig. 4b). IL1B mRNA expression after a 6 day regeneration phase was comparable to the physiological control, (Fig. 4c). AD12d models also showed a characteristic pathological gene expression profile of AD, with strongly elevated mRNA levels of the AD biomarkers CCL26, CA2, and NELL2, (Fig. 4d-f). Following the 6-day regeneration phase without Th2 cytokine stimulation, the mRNA levels of these AD biomarkers were reduced to physiological levels, (Fig. 4d-f).

### Treatment of the 3D AD model with celastrol

**Fig. 5:**
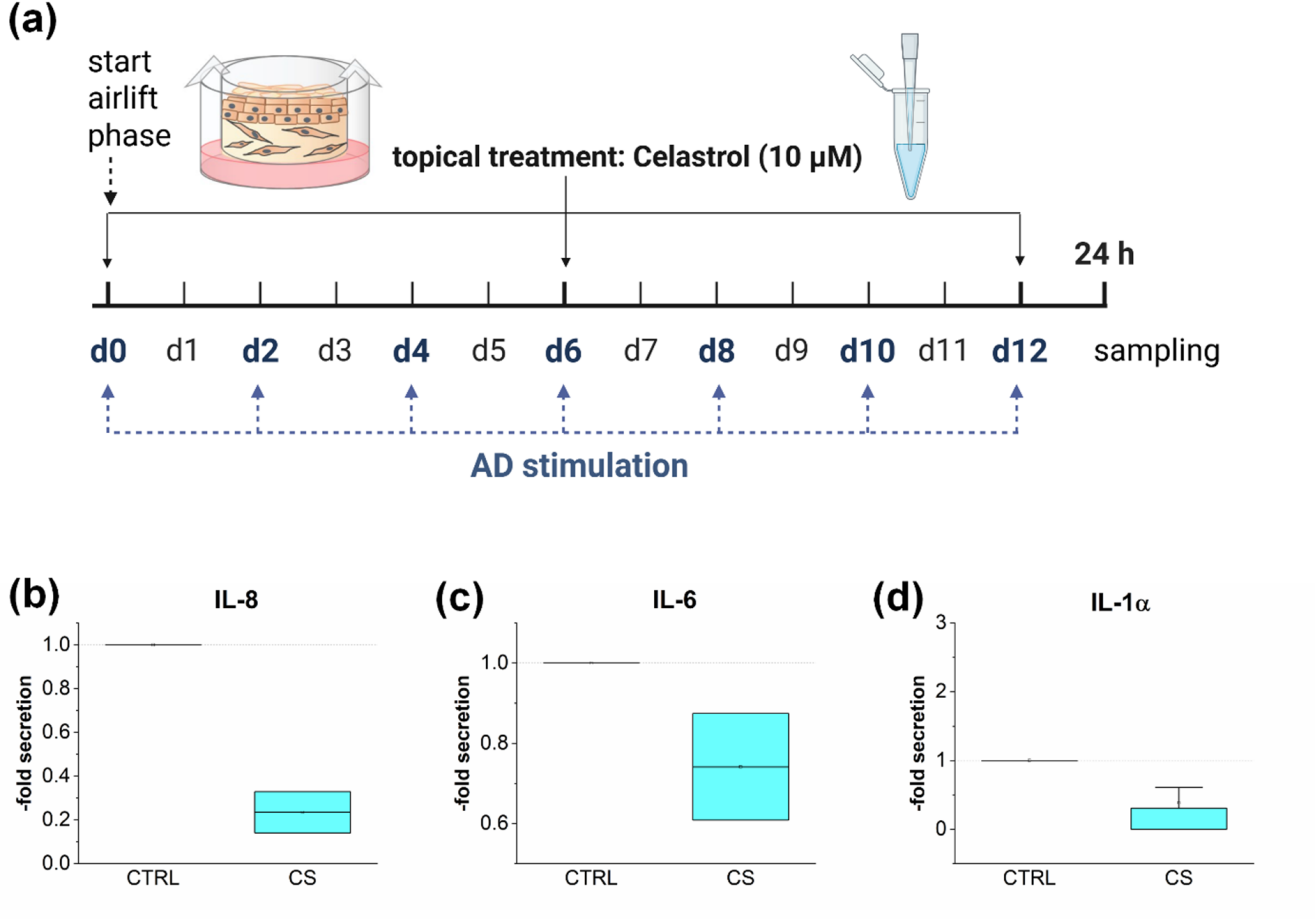
Treatment of 3D AD models with 10µM celastrol (CS) on days 0, 6, and 12 of the airlift period with simultaneous cytokine stimulation on days 0, 2, 4, 6, 8, 10, and 12 of the airlift phase (**a**). Sampling was done 24 h after last treatment with determination of IL-8 (**b**) IL-6 (**c**), and IL-1α (**d**) in the supernatants using ELISA-kits. CS treated models were compared to vehicle treated AD disease models (CTRL). Created in BioRender. Reddersen, K. (2025) https://BioRender.com/8ygat8t.

To investigate the anti-inflammatory potential of celastrol in the 3D inflammation model of AD, the skin models were topically treated with 10 µM celastrol on days 0, 6, and 12 of the airlift phase, concurrently with AD stimulation using the pathological Th2 cytokine mix (Fig. 5a). This celastrol concentration was not toxic in the AD models (see Supplementary Fig. S1 online). As seen in Fig. 5b-d, celastrol treatment resulted in a reduction of the secretion of the pro-inflammatory cytokines IL-8, IL-6, and IL-1 α compared to the vehicle-treated AD models.

**Fig. 6:**
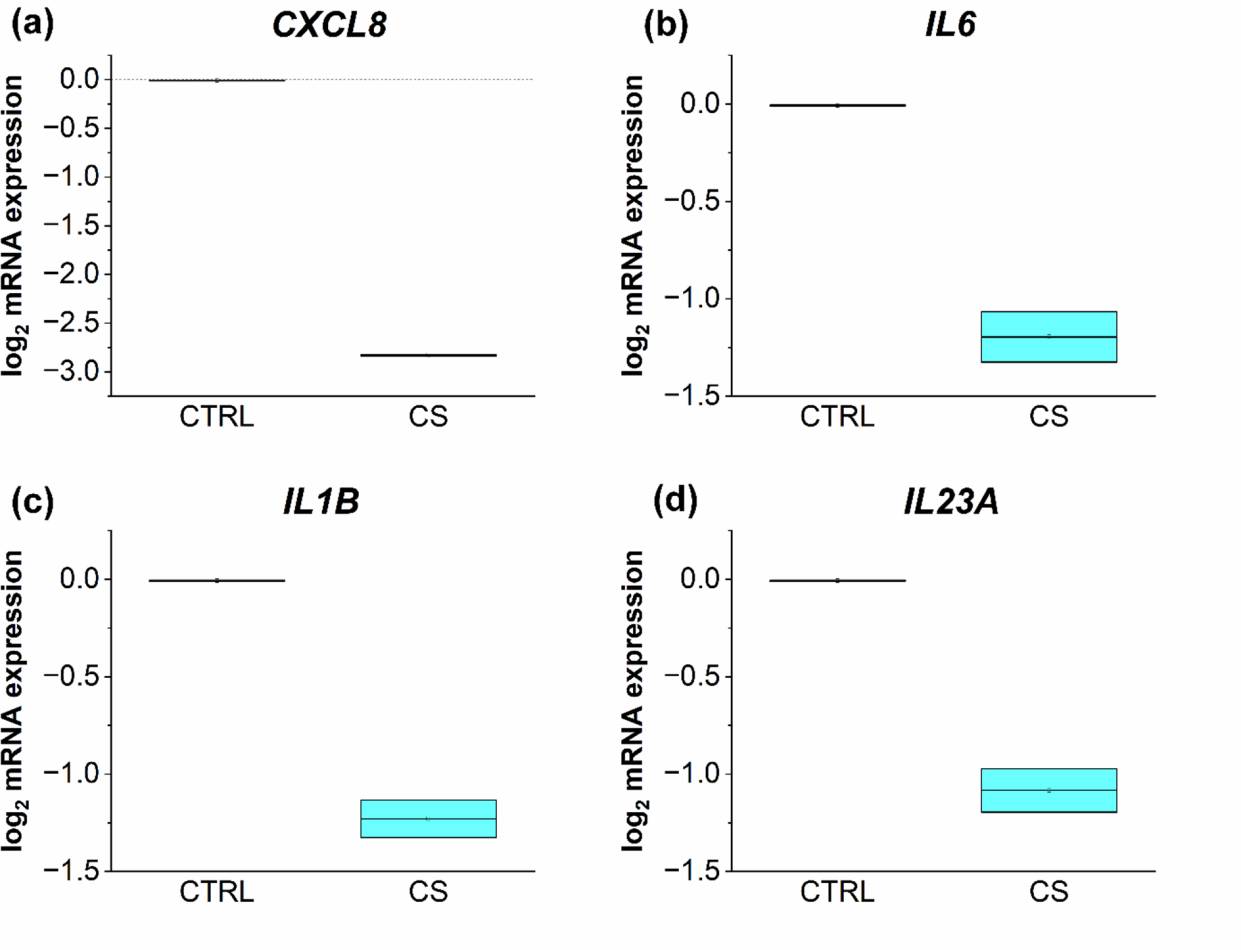
Determination of the relative mRNA expression of the proinflammatory marker *CXCL8* (**a**), *IL6* (**b**), *IL1B* (**c**), and *IL23A* (**d**) of the 3D AD models treated with 10µM celastrol (CS) on days 0, 6, and 12 of the airlift period (CS) compared to vehicle treated AD disease models (CTRL). Gene expression was measured using RT-qPCR. Values were plotted as log2 changes with log_2_ (2) = 1 presenting a doubled upregulation and log_2_ (0,5)=−1 presenting a downregulation by factor 2 compared to the control.

At the transcriptional levels, treatment with 10 µM celastrol downregulated the mRNA expression of all investigated pro-inflammatory genes, including *CXCL8*, *IL6*, *IL1B*, and *IL23A* in AD models compared to vehicle-treated disease models (Fig. 6a-d).

**Fig. 7:**
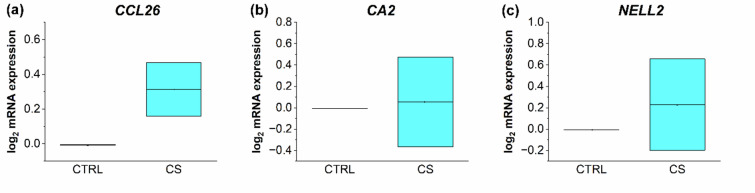
Determination of the relative mRNA expression of the AD markers *CCL26* (**a**), *CA2* (**b**), and *NELL2* (**c**) of the 3D AD models treated with 10µM celastrol (CS) on days 0, 6, and 12 of the airlift period compared to vehicle treated AD disease models (CTRL). Gene expression was measured using RT-qPCR. Values were plotted as log2 changes with log_2_ (2) = 1 presenting a doubled upregulation and log_2_ (0,5)=−1 presenting a downregulation by factor 2 compared to the control.

The mRNA expression levels of the AD biomarker *CCL26*, *CA2*, and *NELL2* were not modulated in a disease-alleviating manner by celastrol treatment. While the *CA2* expression was rather unresponsive to the topical treatment (Fig. 7b), *CCL26* and *NELL2* were even further upregulated by celastrol treatment compared to the vehicle treated AD control (Fig. 7a, c).

## Discussion

To evaluate the anti-inflammatory potential of celastrol in 2D inflammation skin models, HaCaT keratinocytes and primary dermal fibroblasts were stimulated with TNF-α or IFN-γ, to mimic chronic inflammation induced by CD4^+^ T helper 1 (Th1) cells and simultaneously incubated with increasing concentrations of celastrol. Viability of TNF-α- or IFN-γ-stimulated HaCaT keratinocytes decreased at celastrol concentrations of 0.5 and 1 µM. Previously published data on the cytotoxicity of celastrol in HaCaT keratinocytes report comparable cell-compatible concentrations of 1 µM^19^, 2 µM^20^, and 0.7 µM^21^ in non-inflammatory 2D model systems. The cytotoxic effect of celastrol in 2D inflammation HaCaT models has not yet been described. Decreased viability of TNF-α- or IFN-γ-stimulated dermal fibroblasts at 0.5 µM celastrol is consistent with findings in non-inflammatory 2D fibroblast models, where tolerable celastrol concentrations ranged from 0.3 to 2 µM after 24 h of incubation^21–23^.

Stimulation of HaCaT keratinocytes and dermal fibroblasts with TNF-α and IFN-γ induced a strong inflammatory reaction in both cell types, as evidenced by the secretion of the pro-inflammatory cytokines IL-8 and IL-6. Dermal fibroblasts secreted 10–20 times higher cytokine amounts than HaCaT keratinocytes at the same stimulatory trigger. Comparable findings have been reported for IL-1-stimulated keratinocytes and fibroblasts, where fibroblasts secreted 100–1000 times more IL-6 and IL-8 than keratinocytes^24,25^. In HaCaT cells, IL-8 secretion was dominantly increased following TNF-α stimulation, whereas IL-6 secretion was more pronounced after IFN-γ stimulation. In fibroblasts, TNF-α stimulation triggered markedly increased IL-8 and IL-6 secretion, while IFN-γ had minimal effect. In both 2D cell models, celastrol exerted a concentration-dependent anti-inflammatory effect after 24 and 48 h of incubation. The strongest anti-inflammatory impact on HaCaT cells was observed at 0.5 µM celastrol. In fibroblasts, IL-8 secretion after TNF-α stimulation was lowest at 0.1 µM celastrol. The increase in IL-8 levels at 0.5 µM celastrol in fibroblasts may reflect cytotoxic effects.

A limitation of the present study is the use of immortalized HaCaT keratinocytes instead of primary human keratinocytes. While HaCaT cells allow robust culturing under serum-containing conditions and facilitate direct comparison with primary dermal fibroblasts, they do not fully recapitulate the molecular and functional characteristics of primary keratinocytes. Differences in transcriptional regulation and signaling responses have been reported. Nevertheless, HaCaT cells have been shown to exhibit relevant inflammatory responses when carefully cultured, supporting their suitability for exploratory inflammation studies^26,27^. Future investigations including primary keratinocytes will be essential to further strengthen the translational relevance of the findings.

The anti-inflammatory properties of celastrol are attributed to its ability to modulate cytokine and chemokine production, to regulate inflammatory mediators, and to influence immune cell activities. Celastrol targets various signalling pathways involving pro-inflammatory cytokines and chemokines, immune cells and apoptosis^7^. In the literature, the anti-inflammatory effect of celastrol is described for *in vitro* models of different cell types, including macrophages^15,16,22,28,29^, orbital fibroblasts^23^, and keratinocytes^12,17^. In LPS-induced inflammatory macrophages, celastrol reduced secretion of IL-6 and TNF-α via inhibition of p-38 MAPK/MK2 signalling or by suppressive effects on the activity of AP-1 and MAPK^28,29^. Using macrophage cell models, Pace et al.^15^ demonstrated that celastrol alters the biosynthesis of inflammation-related lipid mediators by modulating the activity of different lipoxygenases (LOXs), especially 5-LOX along with decreased formation of pro-inflammatory leukotrienes. The same group further demonstrated that celastrol affects macrophage polarization and suppresses the formation of pro-inflammatory cyclooxygenase-derived prostaglandin E_2_^16^. Treatment of IL-1ß-induced inflammatory orbital fibroblasts with celastrol has been shown to reduce the expression of IL-6, IL-8, ICAM-1, COX-2, and prostaglandin E_2_ by inhibiting NF-κB activation^23^. In psoriatic 2D keratinocyte models, celastrol demonstrated a potent anti-inflammatory effect following stimulation with imidiquod^12^ or IL-17, oncostatin M, and TNF-α^17^. In both studies, celastrol treatment markedly reduced the expression of pro-inflammatory markers, including IL-6, TNF-α, IFN-β^12^ or IL-8 and IL23A^17^.

The *in vitro* 3D AD model employed in the present study has been previously established and comprehensively characterized in terms of disease development^30^. This earlier work focused on model establishment, while this study focussed on treatment of these models with celastrol, which has not previously been investigated in AD associate *in vitro* models. A critical prerequisite for testing anti-inflammatory therapies in disease models is the evaluation of the capacity of these models to revert to a physiological phenotype. To assess the regenerative potential of the AD models, they were first primed for 6 days with a Th2 cytokine mix, followed by a 6-day regeneration period without cytokine priming during airlift phase. In our experiments, inflammatory parameters rapidly returned to physiological levels during the regeneration phase compared to fully stimulated models (AD_12d_). Inflammation-associated genes like *IL1B* and *CXCL8* showed a clear trend toward physiological gene expression levels at the end of the regeneration phase. In fully stimulated AD_12d_ models, the mRNA level of *IL1B* was notably lower compared to physiological controls. Reports on IL-1ß levels in AD patients are inconsistent. Räsänen et al. described a significantly reduced IL-1 production by epidermal cells from AD patients compared to healthy controls^31^, whereas other studies reported elevated IL1ß levels in atopic individuals^32^. For *IL6*, a reduction of the mRNA levels was observed in fully stimulated AD_12d_ models, and this reduction persisted throughout the 6-day regeneration period. This could be due to the strong inhibitory effect of IL-4, which is part of the Th2 cytokine mix used for disease model induction. IL-4 is a potent inhibitor of *IL1B* and *IL6* mRNA expression and protein secretion^33^. Thus, the downregulation of *IL1B* and *IL6* in our models is likely a direct consequence of Th2 cytokine exposure. At the protein level, the elevated release of the pro-inflammatory mediators IL-8 and IL-1α returned to physiological levels after exposure to regeneration conditions. In case of IL-6 secretion, which was reduced in fully stimulated AD_12d_ models, an increase during the regeneration phase was observed but did not fully reach physiological levels. This effect can most likely be attributed to the sustained influence of IL-4. Furthermore, the elevated mRNA levels of the AD biomarkers *CCL26*, *CA2*, and *NELL2* in AD_12d_ models normalized to physiological levels after the 6-day regeneration period. In summary, the model demonstrated a high degree of reversibility of inflammatory parameters and biomarker expression, confirming its high suitability for use in therapeutic intervention studies.

Celastrol at concentrations of 10 µM was well tolerated in 3D AD models, underscoring the robustness of 3D skin models compared to 2D cell monolayers. In contrast, the cytotoxic threshold in 2D skin cell models is more than ten times lower, namely at 0.5–1 µM celastrol. The complex tissue architecture and the presence of a functional stratum corneum in 3D models enable the use of higher, more clinically relevant concentrations than in the more vulnerable 2D cell monolayer models^34,35^. In the 3D atopic dermatitis model, celastrol was evaluated at a fixed concentration, and no dose–response analysis was performed. This was due to the use of predefined celastrol formulations generated in parallel animal experiments within the same project. As a result, conclusions regarding concentration-dependent effects or therapeutic windows cannot be drawn from the present data. Future studies should address dose–response relationships to further refine optimal topical concentrations.

Topical application of celastrol in the 3D AD model elicited a pronounced anti-inflammatory effect, reflected by markedly reduced secretion of the pro-inflammatory cytokines IL-8, IL-6, IL-1α, as well as the mRNA expression of inflammation-associated genes such as *CXCL8*, *IL6*, *IL1B*, and *IL23A*. IL-1, a biologically active cytokine stored in keratinocytes, is one of the earliest mediators released upon inflammation and triggers the cytokine cascade^36^. It induces other pro-inflammatory mediators like IL-6, IL-8, TNF-α, and GM-CSF, and promotes the recruitment of neutrophils, macrophages, and T cells to the site of inflammation^36^. IL-8, a pro-inflammatory chemokine abundantly produced in atopic skin, has been shown to correlate with disease severity in AD patients^37^. IL-6 is a pleiotropic cytokine with immunomodulatory functions, influencing growth and differentiation of various cells types including hepatocytes, fibroblasts, keratinocytes, monocytes, T cells, B cells, and NK cells^38,39^. Beyond its elevated expression in local lesions, IL-6 is also detectable in the systemic circulation, thus mediating whole-body inflammatory reactions^38^. The pro-inflammatory cytokine IL-23 is involved in activating IL-17-producing immune cells such as T cells, mast cells and neutrophils^40,41^. It is produced by epidermal Langerhans cells, dendritic cells, macrophages, and keratinocytes. Its expression is upregulated in the skin of AD patients compared to healthy individuals^42^.

Our results are consistent with the downregulation of *IL1B*, *IL6*, *CXCL8* and *IL23A* gene expression observed after celastrol treatment in previous *in vitro* and *in vivo* inflammation studies^13,14,17–19,23,43,44^. In addition to 2D cell models, the majority of these studies employed mouse models^12–14,19,45^, with only few investigations using *in vitro* 3D disease models. Among these, the effect of celastrol has so far only been described in psoriasis models^17,18^. This underscores a clear need for the establishment and application of various *in vitro* disease models in preclinical research as an alternative to animal testing in accordance with the 3Rs principle (replace, reduce, refine). Studies investigating celastrol in AD mouse models^14,19^ demonstrated inhibition of several inflammatory mediators, such as IL-1ß, IL-23, TNF-α, and IL17 along with reduced epidermal thickness, mast cell infiltration and decreased scratching level. It has been proposed that in allergen-stimulated keratinocytes celastrol may inhibit the release of the alarmin TSLP, as well as IL-25 and ezrin. This suppression could prevent the initiation of the immune cascade, thereby attenuating the Th2-type inflammatory response driven by IL-4 and IL-13 release^19^.

The molecular mechanisms underlying the observed anti-inflammatory effects were not directly assessed in this study and are inferred based on established literature. Given its classification as a multitarget inhibitor^46^, celastrol has been reported to modulate numerous pro-inflammatory signalling pathways. These include NF-κB, STAT, MAPK, TLR, PI3K-AKT-mTOR, NLRP3, HMGB1, Nrf2, among others^7,8,10,46^. This pleiotropic mode of action may not only enable celastrol to target upstream regulatory nodes such as transcription factors and inflammasome activation, but also to interfere with downstream effector mechanisms like cytokine release, oxidative stress responses, and immune cell recruitment. This range of molecular targets underscores celastrol’s high therapeutic potential as a bioactive ingredient from TCM, making it an attractive candidate for the treatment of chronic inflammatory skin diseases. While the observed cytokine modulation in our study is consistent with these mechanisms, direct pathway-specific analyses will be required in future studies to confirm their involvement in the present models.

The 3D AD models used in this study have previously been shown to express elevated levels of disease biomarker genes like *CCL26*, *CA2* and *NELL2*^30^. During celastrol treatment, mRNA levels of *CCL26*, *CA2*, and *NELL2* were not reduced; moreover, in case of *NELL2* expression even increased. Similar effects were observed by Kamsteeg et al., where treatment of AD skin models with the glucocorticoid dexamethasone did not lead to a reduction in *CA2* and *NELL2* mRNA levels^47^. This persistence of biomarker expression might be explained by the continuous administration of Th2 cytokines during airlift incubation and topical treatment. *In vivo*, anti-inflammatory treatments can also act on immune cells, thereby reducing their inflammatory cytokine production. However, in this *in vitro* model, the Th2 signalling, mimicking the activity of pathogenic Th2 cells, was maintained throughout the experiment, posing as a continuous re-stimulatory impulse that celastrol could not counteract while targeting fibroblasts and keratinocytes. Thus, rather than indicating disease modification, the observed effects of celastrol should be interpreted as a suppression of inflammatory mediators. Moreover, neither CCL26 nor NELL2 have been so far assessed in prior celastrol studies, hence, these observations are unprecedented in the literature regarding celastrol’s action on these AD biomarkers. Paradoxical or compensatory responses may further maintain or amplify inflammatory mediators via alternative pathways such as TWEAK-IL-13–NF-κB signaling in keratinocytes and fibroblasts, enhancing IL-6, IL-8, IL-23, CCL17, and others^48–53^. Although not demonstrated for celastrol, these mechanisms illustrate how certain biomarkers may persist despite broad anti-inflammatory effects.

The missing immune component of AD represents a clear limitation of this 3D disease model. Furthermore, the limited number of biological replicates represents a methodological constraint of this study and restricts statistical power as well as generalizability. Consequently, additional statistical analyses such as post-hoc testing or effect size calculations were not performed to avoid overinterpretation of the data. The study was designed as an exploratory, proof-of-concept investigation to assess feasibility, tolerability, and biological relevance of celastrol in complex human *in vitro* skin models. Despite these limitations, the consistency of the observed effects across multiple cell types, readouts, and model systems supports the internal coherence of the results and provides a rationale for future, adequately powered studies.

In summary, the combined use of complementary 2D and 3D human *in vitro* cell models allowed an exploratory assessment of the anti-inflammatory effect of celastrol. The Th1-driven 2D inflammation models of HaCaT keratinocytes and dermal fibroblasts provided insight into cytokine-specific responses and cytotoxic effects, while the Th2-driven 3D disease model of AD reflected structural and functional complexity of human skin. Although limited by sample size and model-specific constraints, the consistent reduction of inflammatory markers at both transcriptional and protein levels supports the potential of celastrol as a multitarget anti-inflammatory compound. Together, these models demonstrated that celastrol effectively reduces both transcriptional and protein-level markers of inflammation, even under continuous cytokine stimulation. These findings provide a foundation for future studies employing expanded biological replication, primary keratinocytes, and pathway-focused analyses to further evaluate its therapeutic relevance in chronic inflammatory skin diseases.

## Materials and methods

### Cytotoxicity and anti-inflammatory testing of celastrol in 2D keratinocyte and fibroblast inflammation models

Human HaCaT keratinocytes (CLS, Germany, used from passages 15–20) were seeded with a cell number of 5 × 10E4/mL in 96-well plates (Greiner Bio-One, Germany) and cultured for 48 h in DMEM (Pelobiotech, Germany) supplemented with fetal calf serum (10%, FCS, PAN, Germany) and an antibiotic/antimycotic mix (1%, Pelobiotech, Germany) at 37 °C with 5% CO_2_ under humidified atmosphere. Primary human dermal fibroblasts isolated from juvenile foreskin (used from passages 3–5) were seeded with a cell number of 5 × 10E4/mL in 96-well plates and cultured for 48 h in DMEM supplemented with fetal calf serum (2%), recombinant human insulin (5 µg/mL, Pelobiotech, Germany), recombinant human fibroblast growth factor (5 ng/mL, Cellsystems, Germany) and gentamicin (50 µg/mL, Thermo Fisher, Germany) at 37 °C with 5% CO_2_ under humidified atmosphere. Primary fibroblasts were obtained and handled in accordance with relevant guidelines and regulations provided and approved by the Ethics Committee of the Medical Faculty of the Friedrich Schiller University Jena (4739-03/16). Informed consent was obtained from the legal guardians of all the donors of primary fibroblasts in the study. To induce inflammation, cells were cultivated with human TNF-α (20 ng/mL, 7BioScience, Germany) or human IFN-γ (10 ng/mL, 7BioScience, Germany). Simultaneously, cells were incubated with 0 µM, 0.05 µM, 0.1 µM, 0.5 µM, and 1 µM celastrol. A stock solution of celastrol (5mM) was prepared in DMSO (Sigma-Aldrich, Germany), working solutions were diluted in PBS (pH 7.3, Pelobiotech, Germany). Medium without TNF-α and IFN-γ served as unstimulated control. Medium with 0.02% DMSO served as solvent control for celastrol treatment. This corresponds to the DMSO concentration of the highest tested celastrol concentration.

After 24 and 48 h incubation, viability of the keratinocytes and fibroblasts was determined using a luminometric ATP assay according to manufacturer instructions (ATPLite, Perkin Elmer, USA)^54^. Viability values below 70% are considered as cytotoxic. Additionally, cytotoxicity was determined measuring the activity of lactate dehydrogenase (LDH) in the cell supernatants by a colorimetric assay according to manufacturer instructions (Roche Diagnostic GmbH, Germany). Medium without TNF-α and IFN-γ served as unstimulated control. Triton X-100 (0.1%, Sigma-Aldrich, Germany) served as positive cytotoxic control. LDH values above 30% are considered cytotoxic. Quantification of the interleukins IL-6 and IL-8 in the cell supernatants after 24 and 48 h incubation was done using IL-6- or IL-8-ELISA-kits (Mabtech, Sweden). The tests were performed according to the manufacturers’ protocols.

### Construction of the 3D AD model

The 3D AD model was constructed as described previously^30^. Briefly, primary human fibroblasts were seeded at a density of 1.5 × 10E5 cells per insert (1326 cells/mm^2^, 113.1 mm^2^ per insert, 0.4 μm pore diameter, PET, Greiner Bio-One, Germany) and cultured submersed for 21 days. Primary keratinocytes (1.35 × 10E5 per insert, 1190 cells/mm^2^) were seeded on top of the dermal layer. After 7 days of submerse cultivation, differentiation of the epidermis was induced by airlift cultivation. For induction of the disease symptoms of AD in the 3D skin model, airlift cultivation was done with the addition of a Th2 cytokine mix to the medium reservoir underneath the skin equivalent every 2–3 days for a period of 12 days (50 ng/mL IL-4, 50 ng/mL IL-13, 25 ng/mL IL-31, 7BioScience, Germany)^30^.

### Regeneration of the 3D AD model

3D AD models were constructed as described. To investigate the regeneration ability of the disease models, the Th2 cytokine mix was added at days 0, 2, and 4 of the airlift period. On days 6, 8, and 10 models were fed without the Th2 cytokine mix (AD_6d_). The positive disease control was supplemented on days 0, 2, 4, 6, 8, and 10 with the Th2 cytokine mix (AD_12d_). Skin models without Th2 cytokines in the airlift period served as physiological controls (PHYS). Samples were analysed at day 12 of airlift.

### Treatment of the 3D AD model with celastrol

Treatment of the disease models with celastrol (10 µM) was done on days 0, 6, and 12 of the airlift phase with simultaneous cytokine stimulation on days 0, 2, 4, 6, 8, 10, and 12. Treatment with PBS as vehicle served as control. The samples were analysed 24 h after the final treatment. The used celastrol concentration of 10 µM was not toxic in the AD models (data of LDH release of the PBS control and 10 µM celastrol treatment are shown in Supplementary Fig. S1 online).

### Analysis of cytokine secretion

Analysis of the secretion of interleukin-8 and interleukin-6 in the undernatants of the skin models was done using ELISA kits as described before. Interleukin-1α was measured using a IL1α-ELISA-kit (R&D Systems, USA). The tests were performed according to the manufacturers’ protocols.

### Gene expression analysis

RNA of the skin models was isolated and quantified using real time polymerase chain reaction (RT-qPCR) as described previously^30^. In the regeneration experiments, transcription levels are expressed as log2 changes of the physiological control at day 12 of the airlift period with log_2_ (2) = 1 presenting a doubled upregulation and log_2_ (0,5)=−1 presenting a downregulation by factor 2. In the celastrol treatment experiments, values were plotted as log2 changes compared to the untreated AD disease control. Table 1 lists primer sequences used in these experiments.

**Table 1:** Primer sequences used for RT-qPCR.

Target gene	Primer sequence or order ID	Manufacturer
*ACTB*	fw:5‘- TGCCGACAGGATGCAGAAG − 3’ rev:5‘- CTCAGGAGGAGCAATGATCTTGA − 3‘	Eurofins
*IL1B*	fw: 5‘-GGACAAGCTGAGGAAGATGC-3‘ rev: 5‘-TCCATATCCTGTCCCTGGAG-3‘	Eurofins
*IL6*	fw: 5‘-CCACCGGGAACGAAAGAGAA-3‘ rev: 5‘-GAGAAGGCAACTGGACCGAA-3‘	Eurofins
*CXCL8*	fw: 5‘-TTCTAGGACAAGAGCCAGGAAG3’ rev: 5‘-AAATCAGGAAGGCTGCCAAG-3‘	Eurofins
*IL23A*	fw:5‘-GAATCAGGCTCAAAGCAAGTGG-3’ rev:5‘-AGCAACAGCAGCATTACAGC-3‘	Eurofins
*NELL2*	fw:5‘- AGC CAA AAC ATC AGC CAA GC-3’ rev:5‘- TTC CCT TCA TGG TGC AAG TC-3‘	Eurofins
*CA2*	Hs_CA2_1_SG QuantiTect^®^ Primer Assay	QIAGEN
*CCL26*	Hs_CCL26_1_SG QuantiTect^®^ Primer Assay	QIAGEN

### Statistics

Experiments were conducted twice; each sample was measured in four replicates. Due to the exploratory proof-of-concept nature of the study and the limited number of biological replicates, technical replicates were averaged prior to analysis and biological replicates were used as the independent statistical unit. Data are presented as means ± standard deviation. Statistical significance was evaluated using the non-parametric Mann-Whitney-U test (Graphpad Prism 11.0.0 (84), https://www.graphpad.com/scientific-software/prism/). Graphs were prepared using the OriginPro 2025 (OriginLab, 10.2.0188 teaching, https://www.originlab.com/) and Microsoft^®^ Excel LTSC MSO (Version 2408 Build 16.0.17932.20700, https://www.microsoft.com/).

## Supplementary Information

Below is the link to the electronic supplementary material.

Supplementary Material 1

## Data Availability

All outcome data are fully represented by the quantitative data shown in the graphs included in the manuscript. The raw datasets generated and analysed during the current study are available from the corresponding author on reasonable request.
