# Supplementary material for: Celastrol attenuates Th1- and Th2-driven skin inflammation in 2D and 3D in vitro models
Source: Sci Rep. 2026 Apr 2;16:11382. doi: 10.1038/s41598-026-47386-w (PMC13049077; doi:10.1038/s41598-026-47386-w)
Supplement: Supplementary file 1 — Supplementary Material 1 [file 41598_2026_47386_MOESM1_ESM.docx]

Supplementary





**Supplementary Figure S1:** Determination of LDH release in the AD model after treatment with PBS as vehicle control and 10µM celastrol. No increase in LDH release was observed compared to the PBS control indication no cytotoxicity at this celastrol concentration in the AD model.
